# Report of three imported cases of neurocysticercosis in Guadeloupe

**DOI:** 10.1186/s12879-016-2169-8

**Published:** 2017-01-31

**Authors:** R. Blaizot, B. Melot, K. Schepers, M. Nicolas, S. Gaumond, P. Poullain, L. Belaye, A. Lannuzel, B. Hoen

**Affiliations:** 1 Service de Maladies Infectieuses et Tropicales et Inserm-CIC 1424, Centre Hospitalier Universitaire de Pointe-à-Pitre, Pointe-à-Pitre, France; 2 Laboratoire de Microbiologie clinique et environnementale, Centre Hospitalier Universitaire de Pointe-à-Pitre, Pointe-à-Pitre, France; 3 Service d’anatomo-pathologie, Centre Hospitalier Universitaire de Pointe-à-Pitre, Pointe-à-Pitre, France; 4 Service de Radiologie, Centre Hospitalier Universitaire de Pointe-à-Pitre, Pointe-à-Pitre, France; 5 Service de Neurologie, Centre Hospitalier Universitaire de Pointe-à-Pitre, Pointe-à-Pitre, France; 6 Faculté de Médecine Antilles Guyane, Université des Antilles, EA 4537 Pointe-à-Pitre, Guadeloupe France; 7 Sorbonne University, UPMC Univ Paris 06, Inserm, CNRS, UM 75, U1127, ICM, Paris, F-75013 France

**Keywords:** Neurocysticercosis, Prevention and control, Emerging diseases, Epidemiology, Case reports

## Abstract

**Background:**

Neurocysticercosis is endemic in most countries of Central and South America but has rarely been described in the French West Indies. We aimed to better understand the clinical and radiological presentation of our cases.

**Case presentation:**

We report three cases of neurocysticercosis in patients living in Guadeloupe, with different clinical and radiological presentations.

**Conclusion:**

Given the eventuality of autochtonous transmission, the diagnosis should be considered in all patients living in Guadeloupe presenting with seizures.

**Electronic supplementary material:**

The online version of this article (doi:10.1186/s12879-016-2169-8) contains supplementary material, which is available to authorized users.

## Background

Neurocysticercosis (NCC) constitutes the most frequent parasitic infection of the nervous system [1]. NCC is the expression of the neurological stage of infestation by *Taenia solium* pork tapeworm larvae, when humans become accidental dead-end hosts of the parasitic cycle. Disease results from ingestion of *T. solium* eggs shed in the stool of a human tapeworm carrier. The embryos are released from the egg in the intestines after ingestion and cross the intestinal mucosa toward the bloodstream, which carries them to peripheral tissues including the central nervous system (CNS), where they develop into cysticerci [2]. Lesions are predominantly intracranial although there are other predilection sites like ophthalmic localizations and muscular calcifications [2]. Most of the neurological infections are asymptomatic, however when symptoms are present, seizures are the most common manifestation of the disease, although headaches, neurological deficits, intracranial hypertension, cerebrovascular complications are also reported [2].

NCC is endemic in many tropical countries [3–7]. Numerous imported cases have been described in Western Europe and North America [2]. In France, 67 cases have been reported from 1970 to 2013, most of them thought to be imported from endemic areas [8]. In Haiti and bordering areas of the Dominican Republic, seroprevalence of cysticercosis has been estimated to be around 3% [9–11]. To our knowledge, only four cases diagnosed in the French West Indies had been reported previously in the literature [12, 13].

We report on three additional cases of NCC diagnosed over a 2 month period in Guadeloupe, French West Indies, and discuss each particular clinical and radiological presentation.

## Case presentation

Clinical and radiological presentations are summarized in Table 1.

**Table 1:** Clinical, biological and radiological characteristics of the patients

	Patient 1	Patient 2	Patient 3
Origin	Dominican Republic	Haiti	Haiti
Age (years)	25	52	41
Sex (W/M)	W	W	M
Date of diagnosis (and onset of symptoms)	December 2014	January 2015	December 2014
Time in Guadeloupe	1 year	10 years	Several years
Initial symptoms	Generalized seizure	Generalized seizure	Ischemic stroke and tonic-clonic seizures^a^
Number of colloidal cysts	1	5	0
Number of vesicular cysts	1	1	0
Number of calcified cerebral cysts	3	9	30
*T. solium* antibodies in serum (ELISA)	Negative	Negative	Negative
*T. solium* antibodies in CSF (EITB)	Negative	Positive	Not done
Screening for taeniasis in index case	Negative	Negative	Negative
Screening for taeniasis in household	Negative	Negative	Negative
Treatment	Albendazole + Steroids + Valproic acid	Albendazole + Praziquantel + Steroids + Levetiracetam	Surgical removal^a^ + Albendazole + Steroids + Valproic acid
Seizure recurrence in the first 6 months after treatment	Several	Multiple episodes in the first months after tapering steroids	No recurrence

^a^the initial symptoms and the surgery (done to decrease edema) were unrelated to NCC

### Case 1

**Fig. 1:**
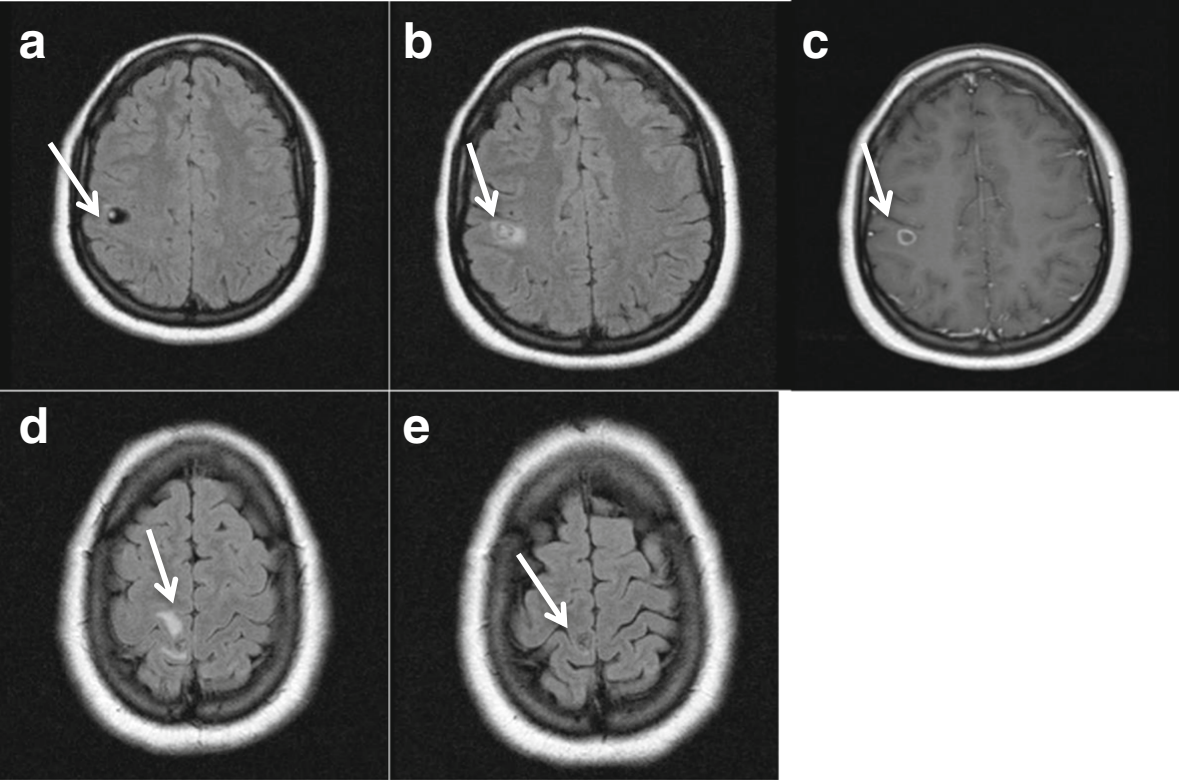
Brain MRI of patient 1 before and after treatment. **a** FLAIR sequence, cysticercus scolex at vesicular stage before treatment; **b** FLAIR sequence, scolex surrounded by edema after treatment; **c** T1 sequence with gadolinium after treatment, single enhancing lesion of scolex at vesicular stage; **d** FLAIR sequence, edema consistent with cysticercus cyst at colloidal stage before treatment, **e** FLAIR sequence, persisting lesion without edema after treatment

A 25-year-old Dominican woman with no medical history was admitted to the department of infectious diseases at Pointe-à-Pitre University hospital with tonic-clonic generalized seizures. She had been living in Guadeloupe for 1 year, had never left the Dominican Republic before moving to Guadeloupe and had never had any focal neurological sign nor seizure before. Brain computed tomography (CT) showed multiple brain lesions consisting of calcified cysts and magnetic resonance imaging (MRI) showed one postero-frontal cyst sheltering a scolex at vesicular stage (Fig. 1a), which fulfilled the diagnosic criteria for definite NCC [14, 15]. Another lesion was surrounded by parenchymal edema, reflecting degenerative changes observed in NCC at the colloidal stage (Fig. 1d). Fundus evaluation was normal and no extraneural localization was clinically found. X ray of legs and arms showed no extraneural “cigar shaped calcification” in muscles or soft tissues. The detection of anticysticercal antibodies by serum enzyme-linked immunosorbent assay (ELISA) (EIA IBL-Diasorin®) was negative. CSF analysis showed no abnormality (2 WBC/mm3, normal proteins = 0.46 g/L, normal glucose level = 3.3 g/L in CSF and 4.3 g/L in blood, negative bacterial culture) and enzyme-linked immunoelectrotransfer blot (EITB) assay for detection of antibodies to *T. solium* glycoprotein antigens on CSF was negative (LDBIO diagnostics®). Stool specimen examination for *T. solium* eggs by microscopy was negative for our patient and all members of her household. No EEG was performed before treatment. She was treated with antiepileptic (valproic acid 500 mg twice daily) and anti-parasitic therapy (albendazole 400 mg twice daily for 14 days) with an adjuvant 14-day dose-reducing course of corticosteroids (dexamethasone 7 mg (0.1 mg/kg) a day for 3 days, then prednisone 70 mg a day for 7 days, then 30 mg for 4 days) [16]. The patient suffered from several focal seizures (clonic movement of the lower leg without impairment of consciousness) with no focal neurological deficit after tapering out cortisone and a generalized seizure 6 month after initiation of treatment. Control MRI at 6 months showed degeneration of the cysts (Fig. 1b, c and e), control EEG was normal but valproate dose was increased to avoid further seizures. At 9 months of follow-up, neurological examination was normal without further seizure activity with 2000 mg per day of valproic acid.

### Case 2

**Fig. 2:**
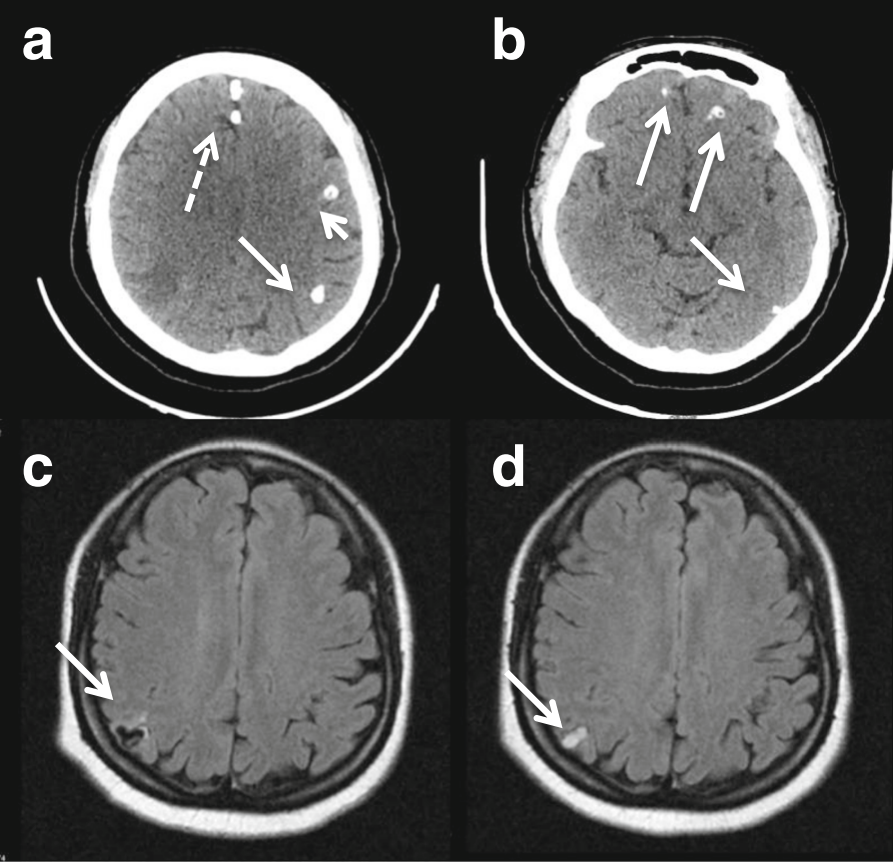
Brain CT and MRI of patient 2 before and after treatment. **a** and **b** Brain CT, multiple calcified cysts before treatment with two probably physiological falx calcifications (discontinued arrow); **c** MRI, FLAIR sequence, cysticercus scolex at vesicular stage before treatment; **d** FLAIR sequence, cyst replaced by edema after treatment

A previously healthy 52-year-old Haitian woman was admitted to the department of infectious diseases of our institution with a generalized tonic-clonic seizure and transient stupor with no focal neurological deficit. She had never had any seizure before but complained of recurrent headaches. She had been living in Guadeloupe for 10 years and had never traveled abroad before and after moving from Haiti. She had no precipitating factor for seizures. CT of the brain showed multiple nodular calcifications in both hemispheres (Fig. 2a and b). MRI showed one right parietal cyst at vesicular stage with vasogenic edema (Fig. 2c) and various at colloidal stage, which fulfilled the diagnosis criteria for definite NCC [14, 15]. Fundoscopy was normal. Extraneural localisations were excluded by X ray of legs and arms. The detection of anticysticercal antibodies by ELISA (EIA IBL-Diasorin®) was negative on serum. CSF biochemical, cytological, and microbiological examinations were normal (no WBC, normal protein level (0.22 g/L), normal glucose level (3.7 g/L in CSF versus 4.4 g/L in blood), negative bacterial culture) but EITB for detection of antibodies to *T.solium* glycoprotein antigens performed on CSF (LDBIO diagnostics®) was positive for anti-p23-26 and anti-p39 antibodies. No evidence of taeniasis was found by microscopy in any of the stool specimens from the patient or her son.

She was treated with a combination of albendazole (400 mg twice daily for 14 days) and praziquantel (1 g three times daily) for 14 days, with dexamethasone started on the same day (6 mg a day for 3 days, then prednisone 40 mg a day for 3 days then 20 mg a day for 4 days) and levetiracetam (500 mg twice daily). A few weeks after tapering out corticosteroids she showed symptoms of focal seizures with daily paresthesia of her left foot and hand that resolved a few weeks after increasing levetiracetam to 1500 g a day. Control MRI after 3 months of treatment showed edema replacing the right parietal cyst. Calcified and colloidal lesions remained stable (Fig. 2d), control EEG was normal.

### Case 3

**Fig. 3:**
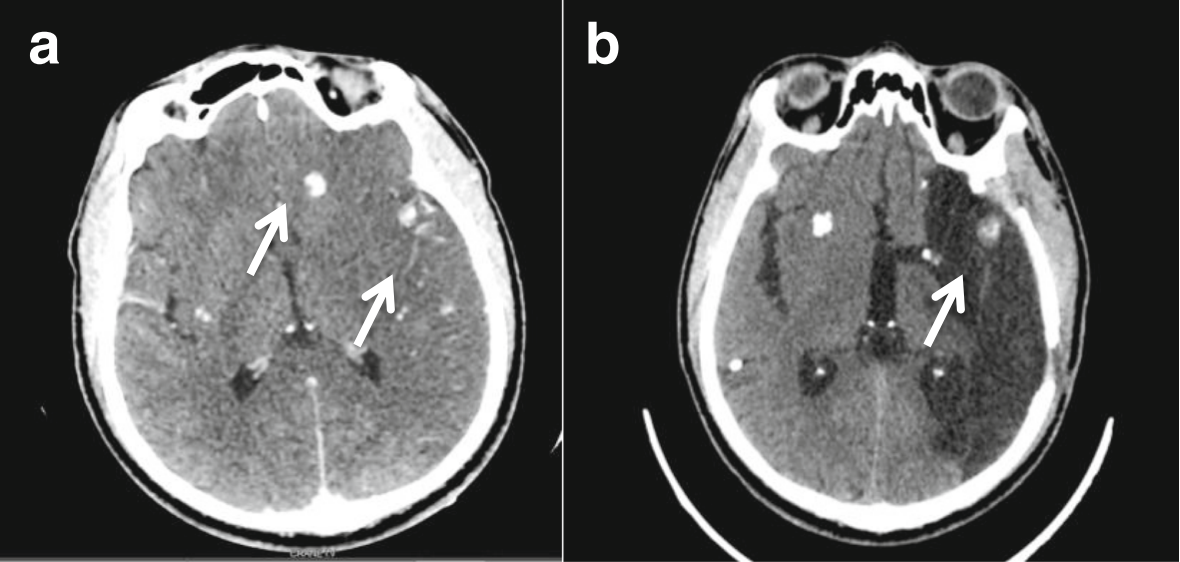
Brain CT of patient 3 before and after treatment. **a** Contrast-enhanced CT, calcified parenchymatous and subarachnoid cysticercus cysts within ischemic territory; **b** Calcified cysts within ischemic territory after craniectomy

**Fig. 4:**
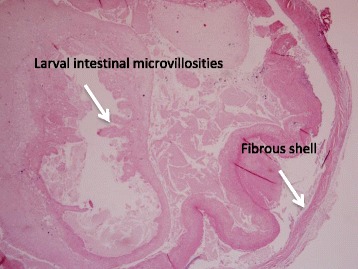
Cross section of a cyst extracted from the brain of the patient 3, stained with H and E at × 40 magnification

A 41-year-old Haitian male with no medical history was admitted to the department of neurology of our institution with right sudden hemiplegia, aphasia and fever and a possible tonic-clonic seizure witnessed by his family. He had been living in Guadeloupe for several years and had not travelled abroad before and after moving from Haiti. He had no previous medical history, in particular no risk factor for stroke. Brain CT showed a left middle cerebral artery infarct secondary to an occlusion of the left internal carotid artery with mass effect. It also revealed multiple supratentorial intraparenchymal and subarachnoid hyperdense homogenous round shaped and infracentimetric lesions in the parietal, frontal, temporal and occipital lobes, consistent with calcified NCC (Fig. 3a). No active lesion was found by CT-scan nor MRI. Duplex ultrasound of supra-aortic vessels showed no cervical arteriosclerosis, the distal left internal carotid was not visible and the signal of the common carotid artery was more restrictive (0.81 vs 0.64) which was consistent with an obstruction. Transcranial duplex ultrasound showed increased velocities of the left median (111 cm/s), posterior (174 cm/s) and anterior (101 cm/s) arteries and of the right anterior (158 cm/s) and posterior (114 cm/s) arteries consistent with the presence of a posterior communicating artery or with angiitis. The left anterior cerebral artery had a retrograde flow, which was consistent with the presence of a posterior communicating artery. CT- angiography (cf Additional file 1) showed an expanded diameter of the internal carotid artery probably due to a thickening of the arterial wall and irregularity of the lumen on its initial portion consistent with a dissection. No fat saturated T1 axial image was performed on MRI to confirm the dissection. Left craniectomy was performed to decrease intracranial hypertension due to cerebral ischemia. During craniectomy, no ischemic tissue was excised but two subarachoidal cysts were discovered and removed. Pathological examination of one cyst showed a round-shaped infracentimetric (0.8× 0.3 cm) structure made of a fibrous shell sheltering an invaginated scolex with intestinal microvillosities and eosinophilic infiltrate consistent with colloidal stage larvae (Fig. 4) which fulfilled the diagnosic criteria for definite NCC [14, 15]. No PCR for *T. Solium* was performed on the cyst. Fundoscopy was normal. No extraneural lesion was found on X ray of legs and arms. The detection of anticysticercal antibodies by serum ELISA (EIA IBL-Diasorin®) was negative and CSF was not tested. Stool examination for *T. solium* by microscopy was negative in the patient but screening of members of his household could not be performed.

Following craniectomy, the patient partially recovered from neurological damage with persisting spastic right hemiplegia and both receptive and expressive aphasia. He was given a 14-day regimen of albendazole (400 mg bid) starting 10 days after surgery, when CT-scan showed a resolution of cerebral edema. This choice was justified by the presence of active lesions inside of the stroke as demonstrated by the presence of living larvae at pathological examination [16]. This treatment was administered along with corticosteroids (1 mg/kg/day for 3 days, rapidly tapered during 6 days) and valproic acid (500 mg twice daily), with no subsequent recurrence of seizures.

## Discussion

Our three cases illustrate three distinct clinical stages and a variety of radiological presentations of the disease (Table 1). All of them were foreigners, coming from areas of higher endemicity, they all had only intracranial manifestations with variability in the number of cysts, and with seizures in the first two cases and probably in the third case.

Our three patients all fulfilled absolute or major clinical, radiological and/or serological criteria for NCC: histological demonstration of the parasite (Patient 3), evidence of cystic lesions showing the scolex (Patient 1 and 2), positive CSF immunoblot for the detection of anticysticercal antibodies (Patient 2), and resolution of intracranial cystic lesions after therapy with albendazole (Patient 1 and 2) [14, 15]. Real time PCR (Polymerase chain reaction) for *T. solium* in CSF can be helpful when the diagnosis is uncertain (sensitivity 83.3%, specificity 100% [17]) but we did not have to perform it because our patients all fulfilled criteria for definite NCC.

Their clinical presentation was slightly different from previous case reports from Guadeloupe [12, 13]. To date, only four other cases have been reported in Guadeloupe in the late nineties. Three of them had intracranial parenchymatous NCC and one had cysticercosis outside CNS, all confirmed by either serology, pathognomonic images on CT scan [12] or histology [13] (MRI was not avalaible at that time in Guadeloupe). Three (1 man, 2 woman) had seizures like our cases, among them one woman had subacute meningitis (180 cells/mm3 in CSF and proteins = 0.6 g/L). The last one (child) had a periorbital intramuscular nodule. Two of them were middle-aged women originating from Haiti where they were probably contaminated as for two of our cases. The man, who was born and lived in Guadeloupe, might have been infected abroad as it was reported that he traveled frequently, and the child had probable autochtonous transmission because he had never left Guadeloupe [12, 13]. More information is missing to better compare them to our cases.

Cerebrovascular and particularly ischemic complications of NCC, are well documented (between 4% and 12% of patients with NCC according to different studies [18, 19]). Chronic subarachnoid inflammation creates angiitis with progressive stenosis, sometimes leading to the occlusion of one or several cerebral arteries [20, 21]. However in patient 3, we cannot draw any causal relationship between the stroke and NCC. The stroke was most probably rather due to the dissection of the left internal carotid artery.

All patients with NCC should receive symptomatic treatment, which consists of antiepileptic medication for patients with seizures, analgetics for patients with pain and corticosteroids for patients with increased intracranial pressure or encephalitis [2]. Antiparasitic treatment should be adapted based on findings from neuroimaging [2, 16], including the localization, number and stage of cysts, and the presence of intracranial hypertension. Different options exist including no antiparasitic treatment, albendazole, praziquantel or both in combination with corticosteroids, and surgical treatment.

In patients with only calcified lesions, antiparasitic treatment has not shown any benefit [16]. For patient 3, despite the presence of only calcified lesions on CT scan, we decided to treat with albendazole, because of the presence of living larvae at pathological examination. In patients with active cysts, albendazole in combination with corticosteroids offers a reduction in the number of generalized seizures in the years following initiation of treatment and the duration of seizure risk compared with placebo [22, 23].

The association of both albendazole and praziquantel has recently shown to increase the parasiticidal effect in patients with multiple viable intraparenchymal cysts [24]. This is why it was used on patient 2.

Patients 1 and 2 experienced several relapses, with partial seizures shortly after treatment initiation despite significant resolution of the cysts as shown on repeat MRI (Fig. 1b, c, e, Fig. 2b and d). The rate of seizure recurrence in the first 12 month after treatment with albendazole is approximately 40% [22, 25]. Inflammation after cyst degeneration with albendazole [2] can be responsible for seizures but is an indicator of efficient cure [26]. Drug-drug interactions between some antiepileptics and albendazole or praziquantel might be a source of antiparasitic treatment failure and a source of persisting seizures [27]. Seizures prior to therapy, persistence of active or calcified lesions on radiological follow-up [22], family history of seizures, serial seizures and electroencephalographic abnormalities [28] have all been described as risk factors for seizure recurrence. There is no clear recommendation in the length of treatment with steroids, but patient 1 and 2 could have benefitted from a longer course on the seizure recurrences.

All three patients had multiple lesions, in common with most cases in adults from endemic areas and particularly in South America; a single enhancing lesion is the most frequent finding in children and in India, where the parasitic load is lower [2, 29].

The three cases described here were probably imported. Patient 1 had most likely the most recently acquired the infection, as indicated by her young age, the CT findings [15, 30, 31], and the fact that she lived in an area of NCC endemicity until 1 year before her first symptoms. Patient 2 suffered from a later stage of the disease and had probably contracted it in Haiti more than 10 years before. Patient 3 is a vivid illustration of a disseminated NCC that had remained clinically dormant for a long time.

In endemic areas clusters of swine cysticercosis have been described around human carriers and justify interventions for control and elimination in humans [32]. In high-income countries transmission from pig-to-human is unlikely due to better human hygiene and veterinary standards of the pigs [33]. *T. solium* eggs have never been found in water or soil [8, 9], however NCC has frequently been imported by tapeworm carriers into non-endemic countries [5]. This confirms that humans can be a reservoir of the disease and that cysticercosis transmission does not require the presence of infected pigs [34]. One case of autochthonous acquisition has already been described in Guadeloupe, in a child who had never left the island and presented with a extracerebral form of the disease (periorbital intramuscular nodule) [13]. Clusters of humans with positive *T. solium* serology have been described around tapeworm carriers, but no cluster of cases of NCCs was seen [35]. As epilepsy due to NCC appear late in the history of the infection, it is not routinely diagnosed around tapeworm carriers [2]. Concurrent intestinal taeniasis is reported in around 5% of the patients with cysticercosis [2, 36, 37]. These patients usually present with a more severe cerebral infection due to repeated auto-infection [2].

We failed to identify tapeworm carriage in our neurocyticerosis patients or those in their households, probably because we used direct examination of stool, which is suboptimally sensitive. Specific coproantigen detection by ELISA assays [38] is more sensitive and could more efficiently detect carriers who would be eligible for treatment with niclosamide to prevent transmission [2, 38].

## Conclusion

NCC should be suspected in patients originating from endemic areas who present with headaches, epileptic seizures or neurovascular symptoms.

Given the possibility of autochthonous transmission in Guadeloupe, it should also be considered in patients who have never traveled to endemic areas, especially when they live among communities with a high prevalence of NCC. Those communities could be responsible for a cluster of cases. A cross-sectional survey to determine prevalence of *T. solium* seropositivity could be very usefull to assess the risk of transmission in Guadeloupe.

## Additional file

Additional file 1: Case 3 CT-angiography images. (ZIP 762 kb)

## Abbreviations

CNS: Central nervous system
CT: Computerized tomography
EITB: Enzyme-linked immunoelectrotransfer blot
ELISA: Enzyme-linked immunosorbent assay
MRI: Magnetic resonance imaging
NCC: Neurocysticercosis
PCR: Polymerase chain reaction
