# Supplementary figures and images for: Report of three imported cases of neurocysticercosis in Guadeloupe
Source: BMC Infect Dis. 2017 Jan 31;17:106. doi: 10.1186/s12879-016-2169-8 (PMC5282648; doi:10.1186/s12879-016-2169-8)

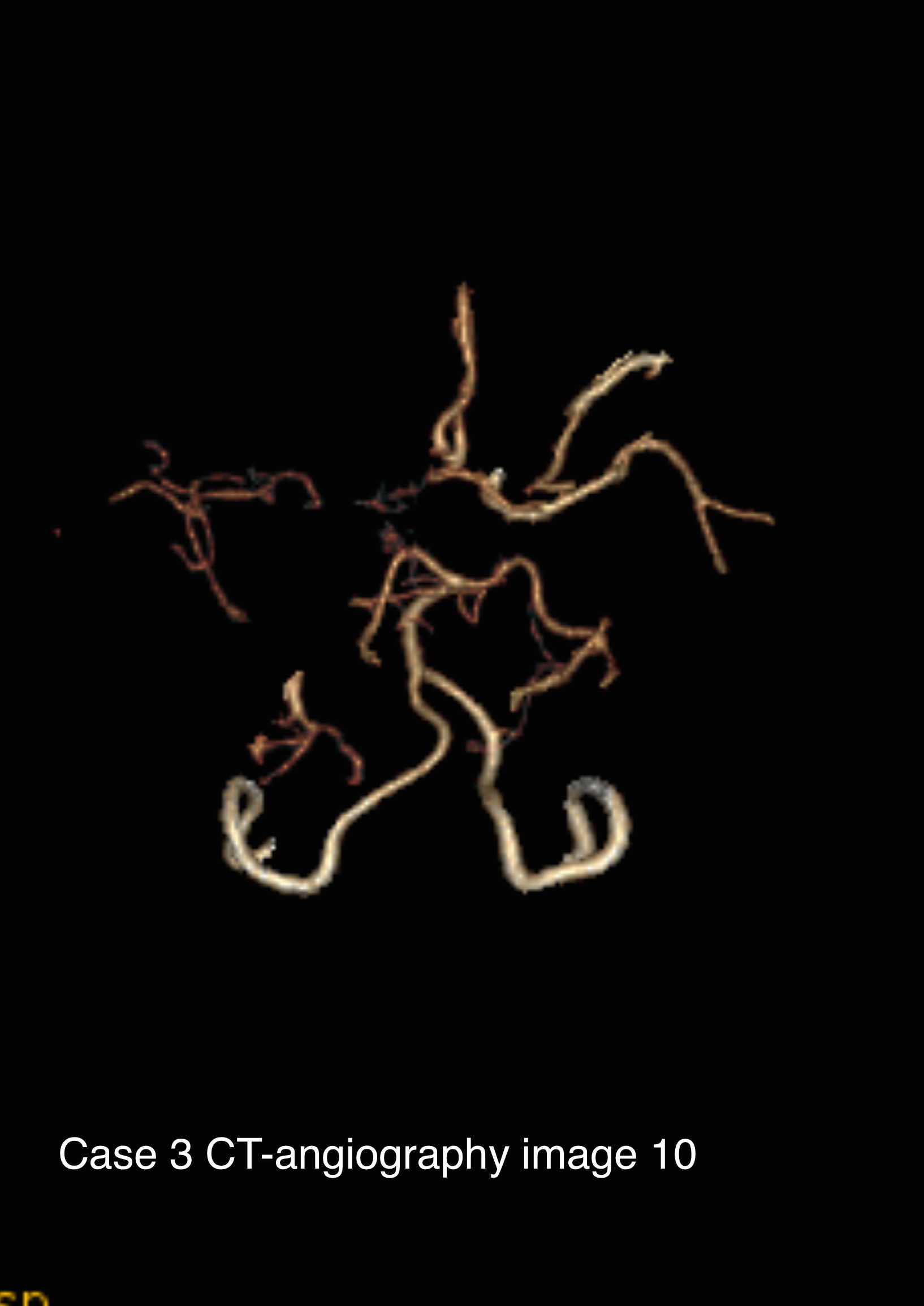

Supplement: Additional file 1: — Case 3 CT-angiography images. (ZIP 762 kb) [file 12879_2016_2169_MOESM1_ESM.zip › additionnal file 10 Case 3 CT-angiography image 10.jpg]

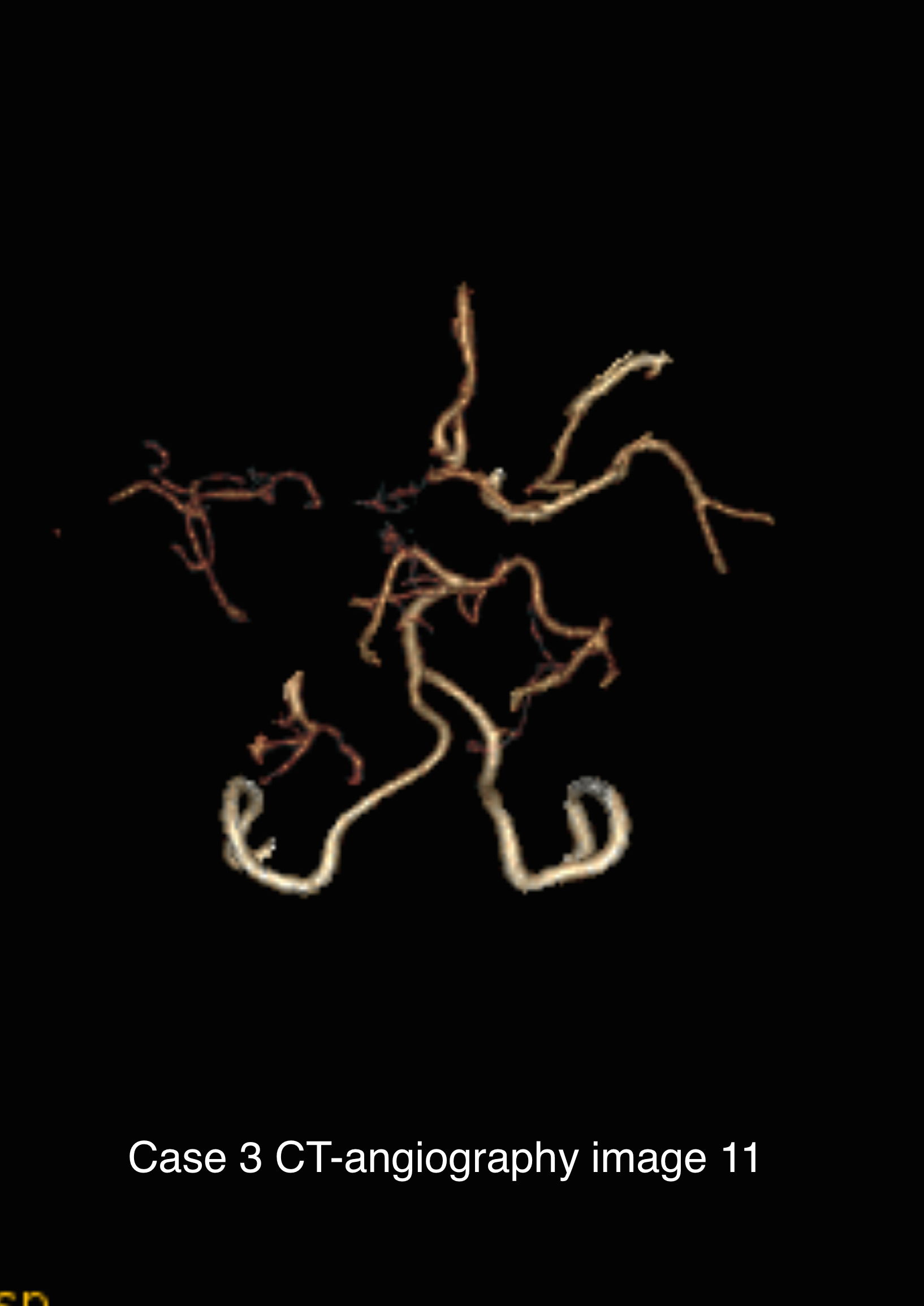

Supplement: Additional file 1: — Case 3 CT-angiography images. (ZIP 762 kb) [file 12879_2016_2169_MOESM1_ESM.zip › additionnal file 11 Case 3 CT-angiography image 11.jpg]

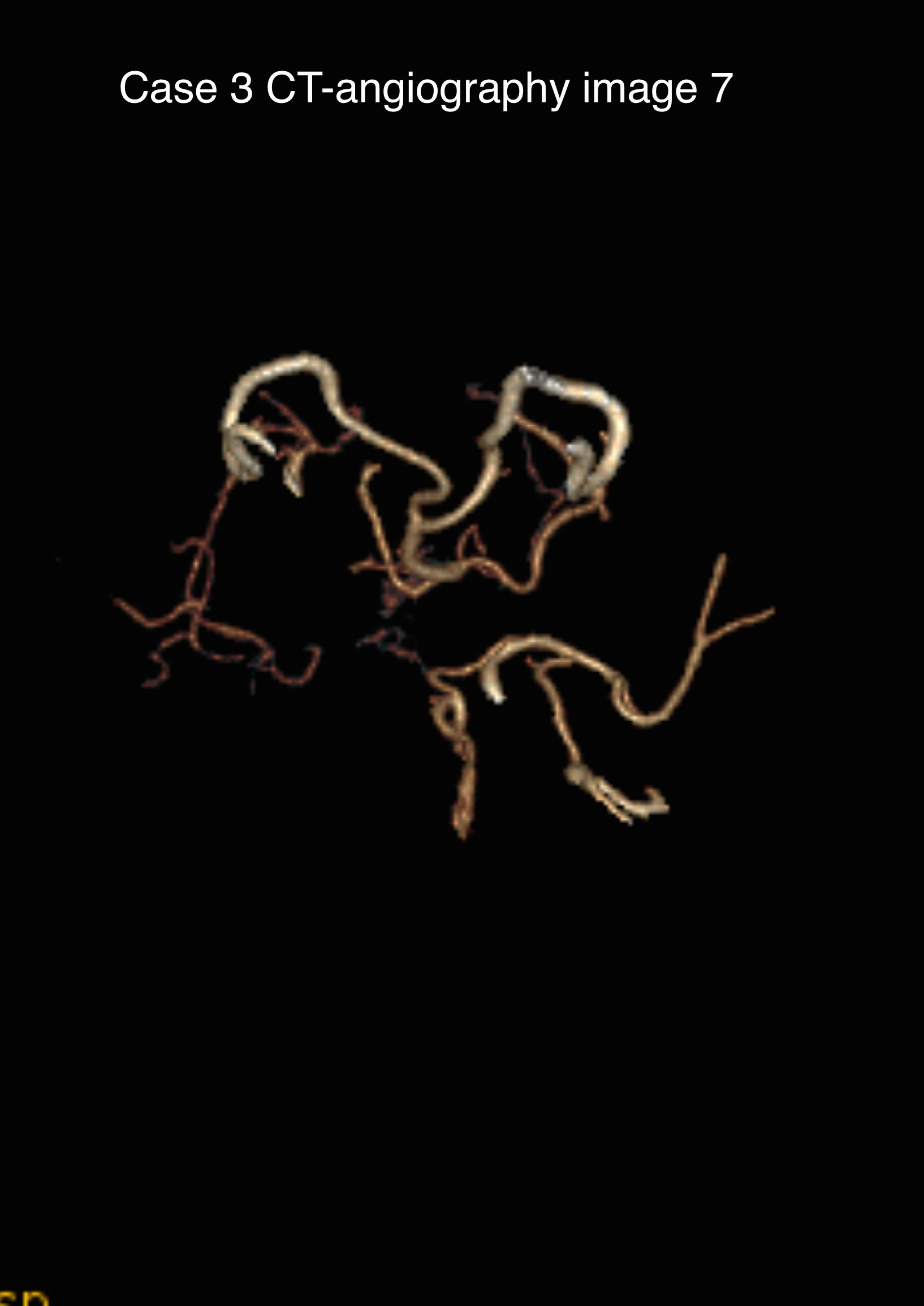

Supplement: Additional file 1: — Case 3 CT-angiography images. (ZIP 762 kb) [file 12879_2016_2169_MOESM1_ESM.zip › additionnal file 7 Case 3 CT-angiography image 7.jpg]

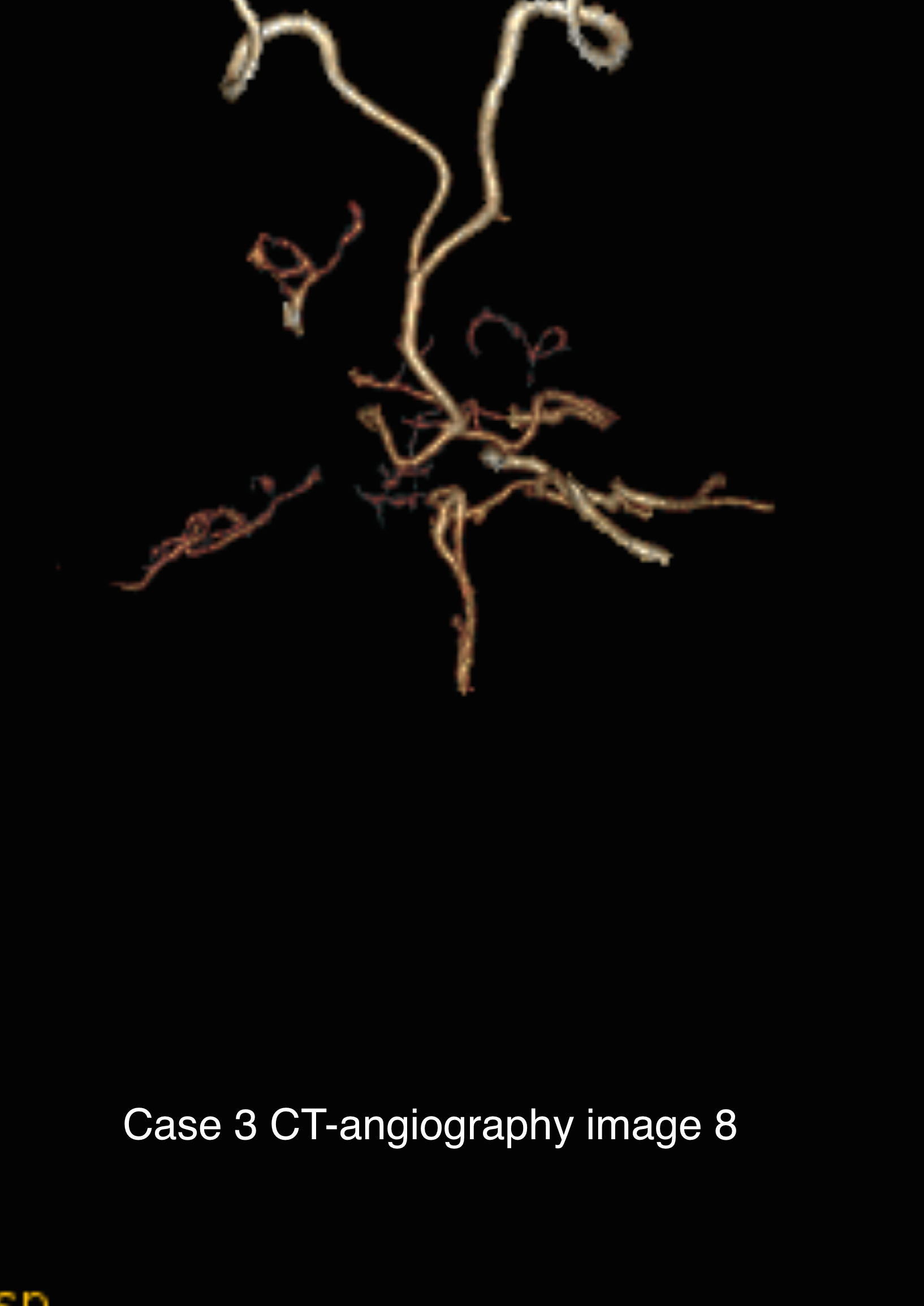

Supplement: Additional file 1: — Case 3 CT-angiography images. (ZIP 762 kb) [file 12879_2016_2169_MOESM1_ESM.zip › additionnal file 8 Case 3 CT-angiography image 8.jpg]

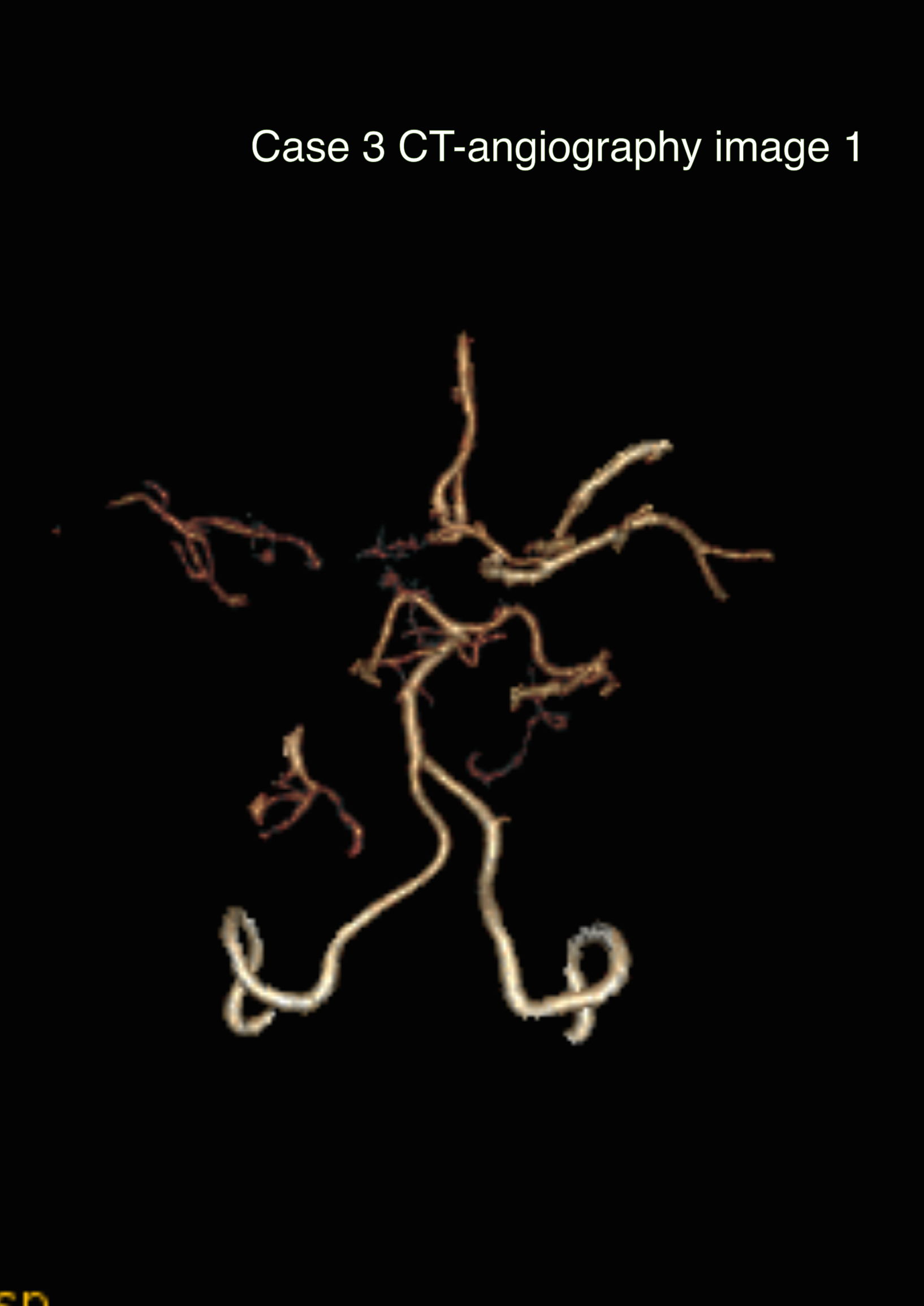

Supplement: Additional file 1: — Case 3 CT-angiography images. (ZIP 762 kb) [file 12879_2016_2169_MOESM1_ESM.zip › additionnal file 1 Case 3 CT-angiography image 1.jpg]

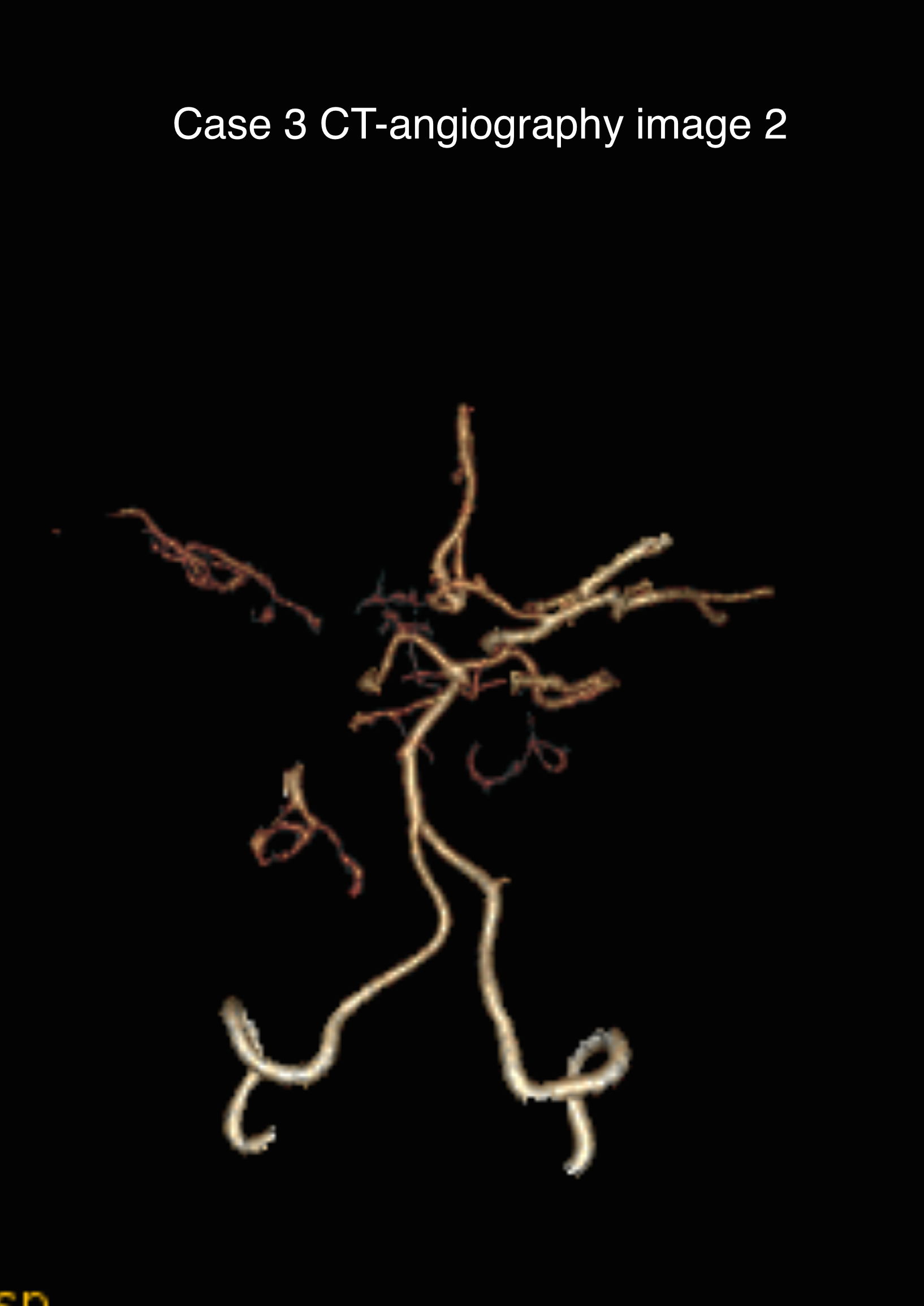

Supplement: Additional file 1: — Case 3 CT-angiography images. (ZIP 762 kb) [file 12879_2016_2169_MOESM1_ESM.zip › additionnal file 2 Case 3 CT-angiography image 2.jpg]

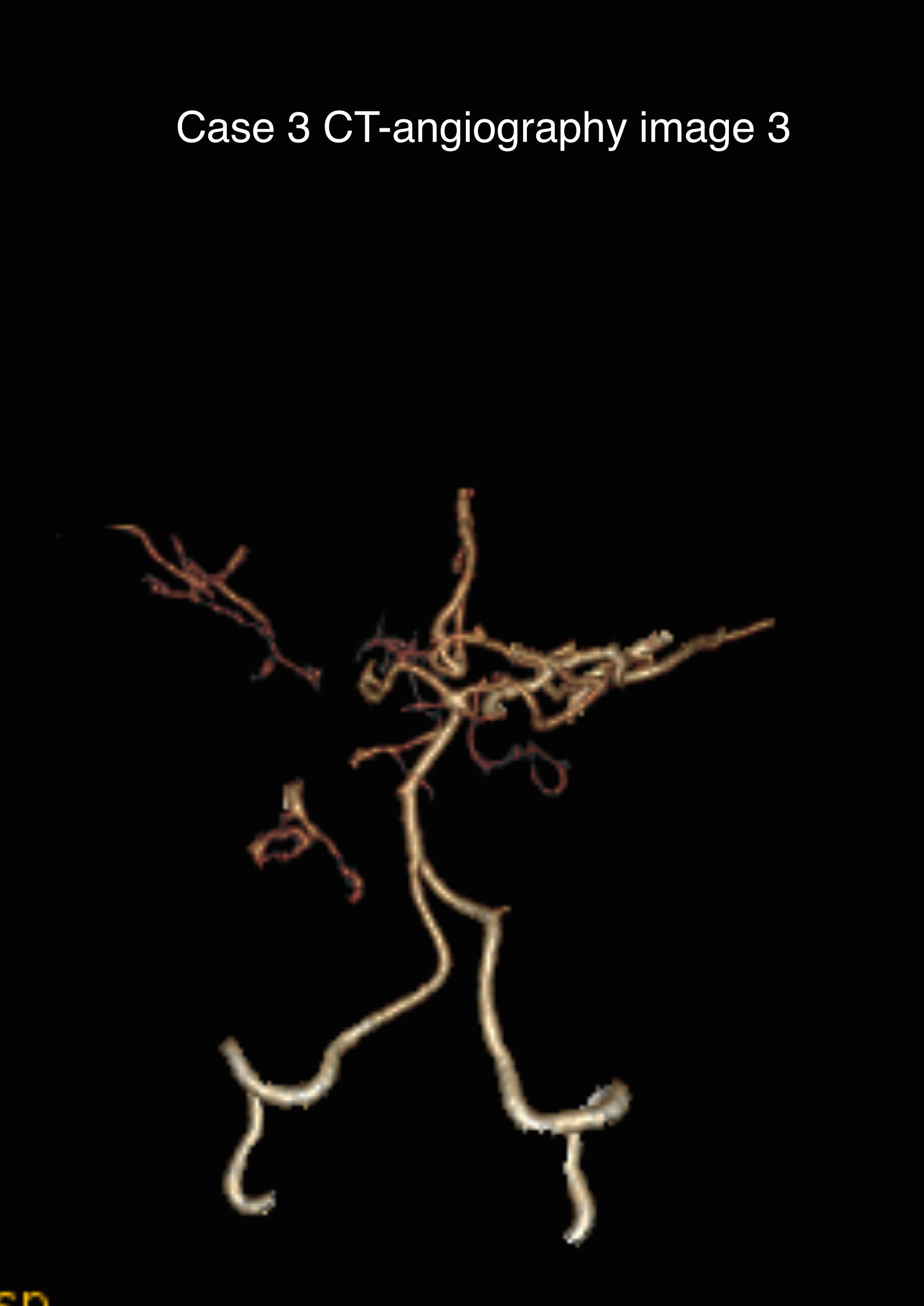

Supplement: Additional file 1: — Case 3 CT-angiography images. (ZIP 762 kb) [file 12879_2016_2169_MOESM1_ESM.zip › additionnal file 3 Case 3 CT-angiography image 3.jpg]

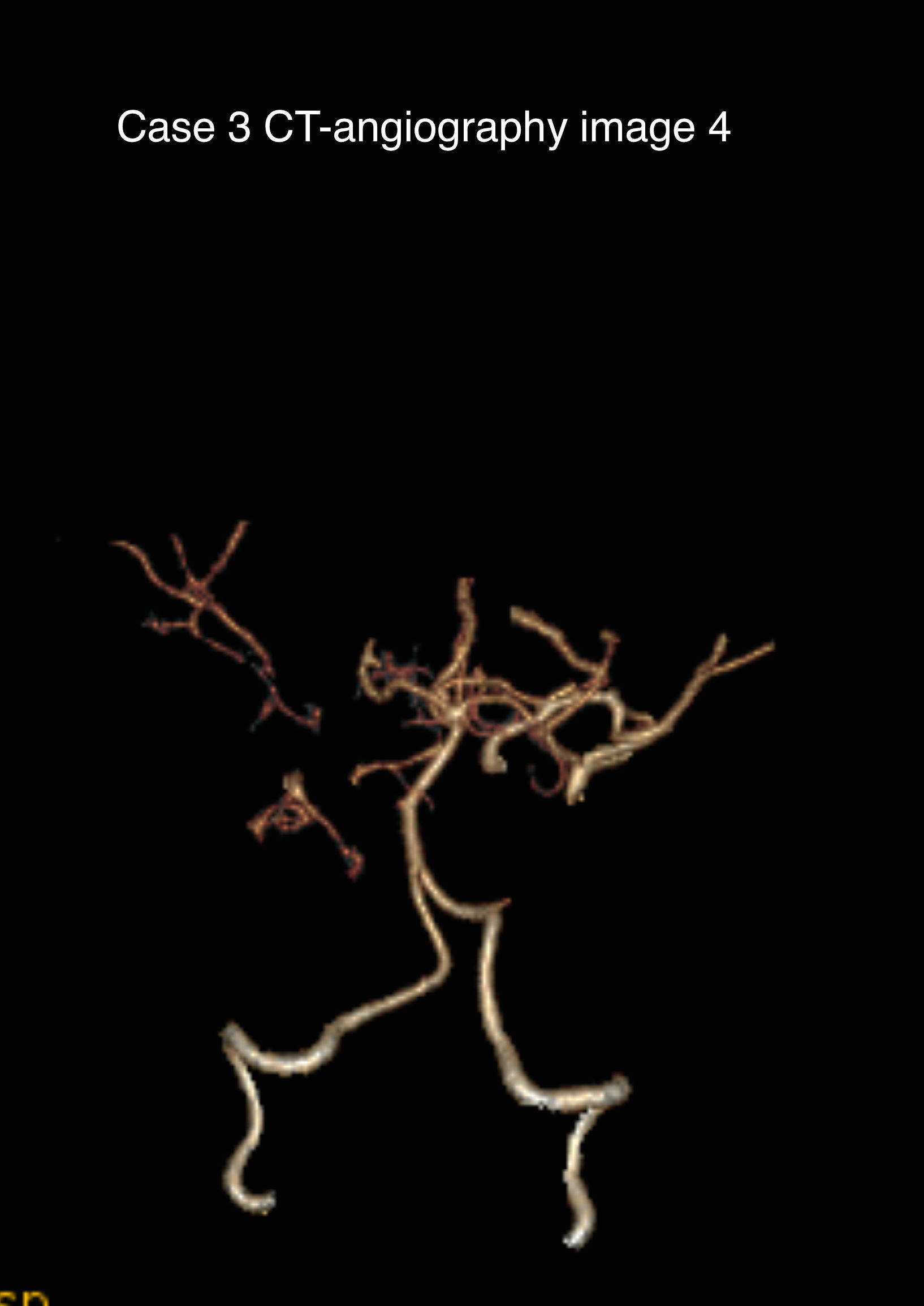

Supplement: Additional file 1: — Case 3 CT-angiography images. (ZIP 762 kb) [file 12879_2016_2169_MOESM1_ESM.zip › additionnal file 4 Case 3 CT-angiography image 4.jpg]

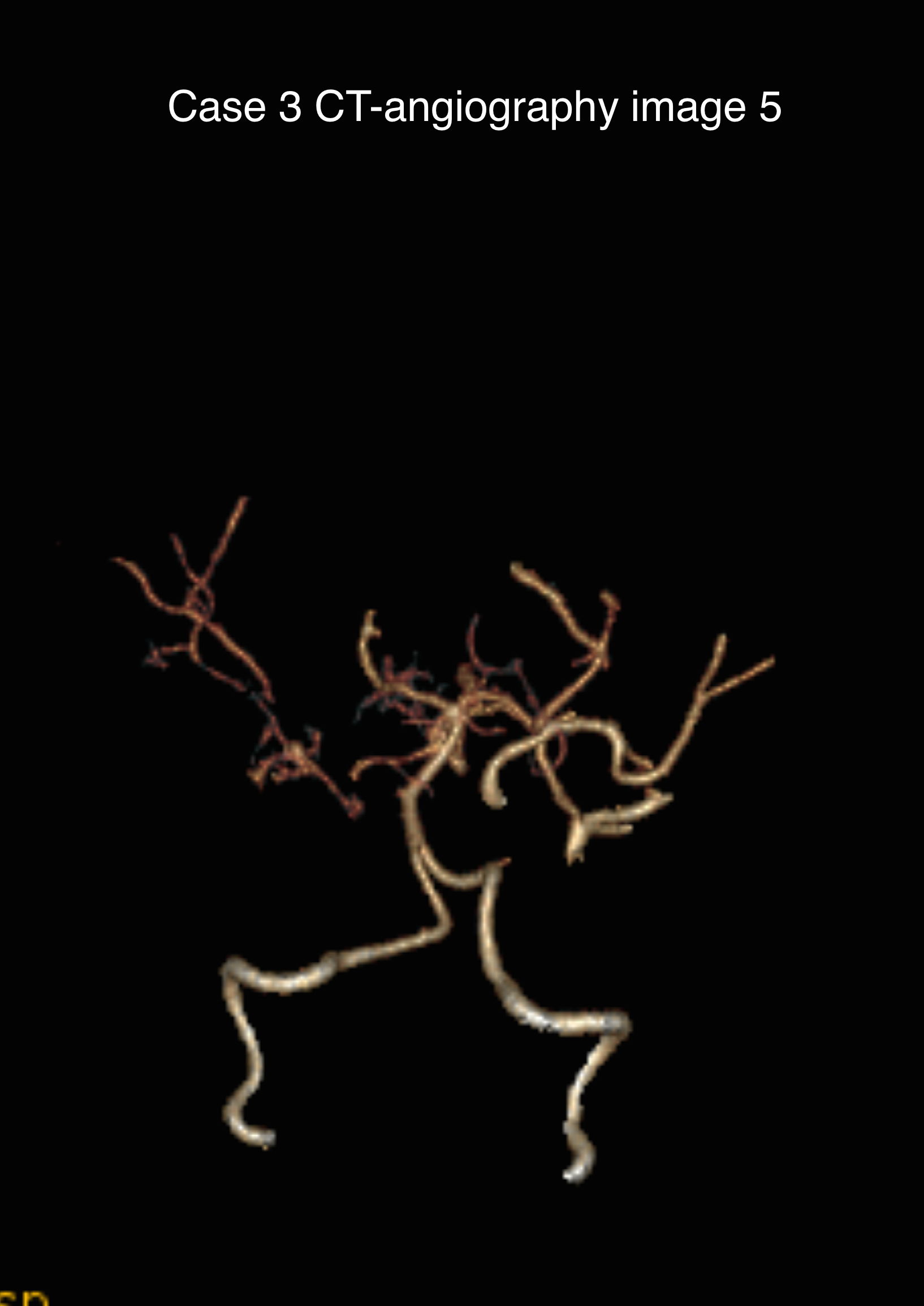

Supplement: Additional file 1: — Case 3 CT-angiography images. (ZIP 762 kb) [file 12879_2016_2169_MOESM1_ESM.zip › additionnal file 5 Case 3 CT-angiography image 5.jpg]

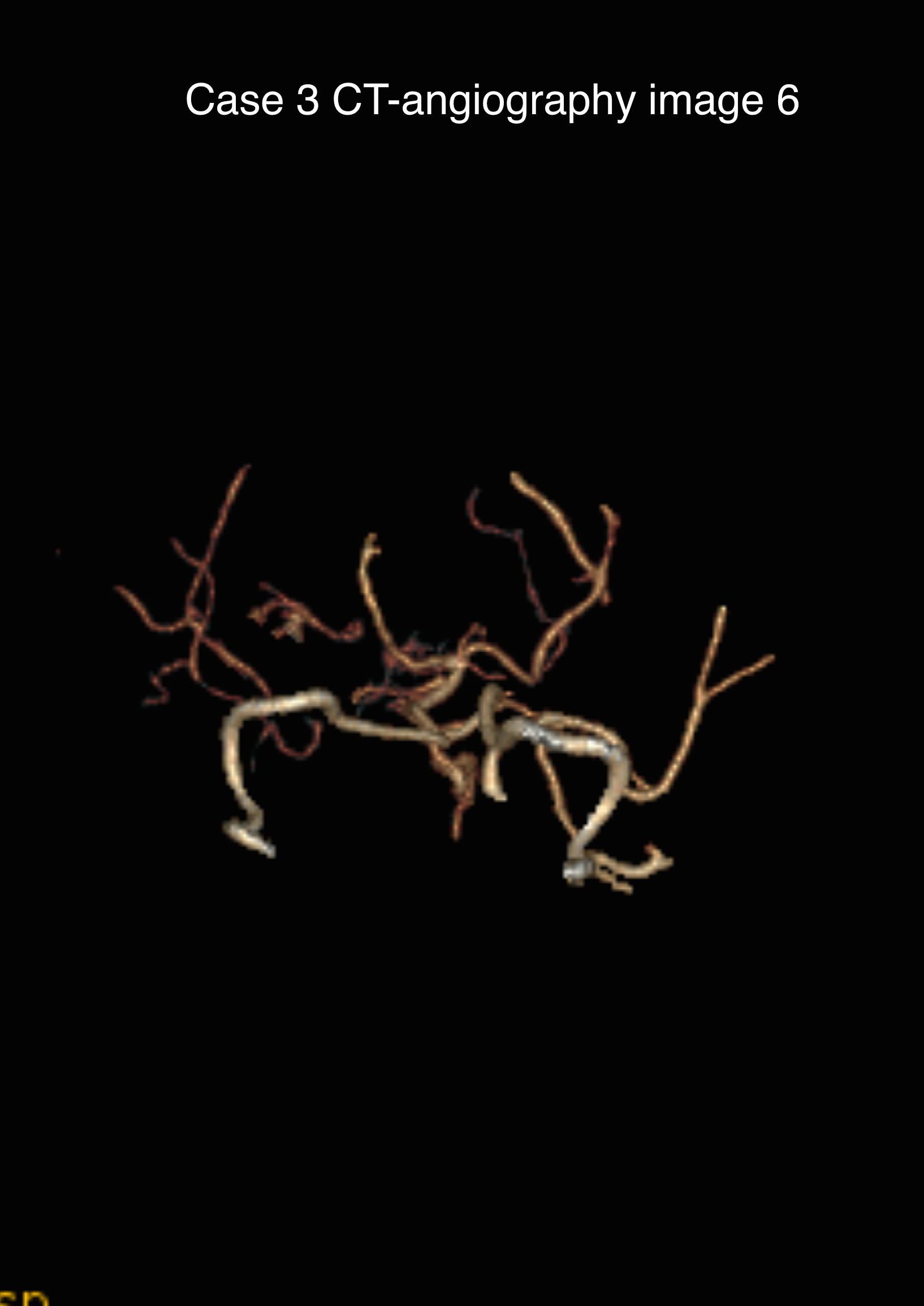

Supplement: Additional file 1: — Case 3 CT-angiography images. (ZIP 762 kb) [file 12879_2016_2169_MOESM1_ESM.zip › additionnal file 6 Case 3 CT-angiography image 6.jpg]

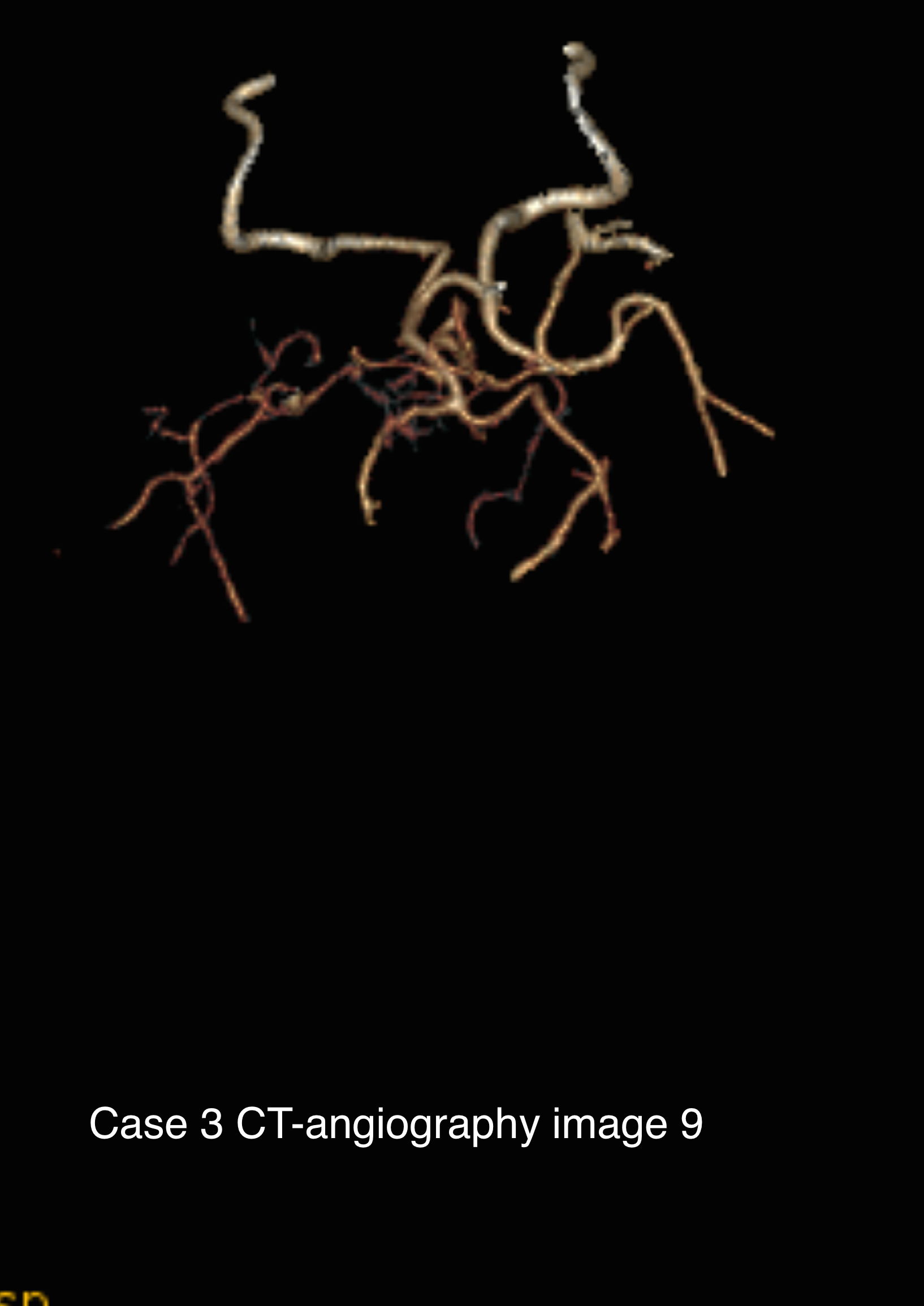

Supplement: Additional file 1: — Case 3 CT-angiography images. (ZIP 762 kb) [file 12879_2016_2169_MOESM1_ESM.zip › additionnal file 9 Case 3 CT-angiography image 9.jpg]
